# BIRDs (Brief Potentially Ictal Rhythmic Discharges) watching during EEG monitoring

**DOI:** 10.3389/fneur.2022.966480

**Published:** 2022-08-23

**Authors:** Ji Yeoun Yoo

**Affiliations:** Department of Neurology, Icahn School of Medicine at Mount Sinai, New York, NY, United States

**Keywords:** seizure, status epilepticus, critical care, drug resistant epilepsy, seizure onset zone, paroxysmal fast activity

## Abstract

Brief Potentially Ictal Rhythmic Discharges (BIRDs), initially described in neonates, have been shown to correlate with increased risk of seizures in both critically ill and non-critically ill adults. In critically ill patients, BIRDs are associated with acute brain injury and worse functional outcomes. In non-critically ill adults, BIRDs are seen in patients with epilepsy with a greater likelihood of having drug resistance. The location of BIRDs seems to better predict the seizure onset zone compared to other interictal epileptiform discharges. The definition of BIRDs includes Paroxysmal Fast Activity (PFA), and they have similar clinical significance regardless of the exact cut-off frequencies. Their potential as a biomarker for seizure activity and seizure onset zone has been suggested. In patients with status epilepticus, BIRDs also resolve or decrease when seizures resolve. Thus, if BIRDs are observed on scalp EEG, more extended EEG monitoring is recommended to estimate their seizure burden and to guide treatment. With the recent addition of BIRDs in the critical care EEG terminology, with future investigations, we may soon be able to reach a consensus about the definition of electrographic seizures and better understand their neurophysiology and clinical significance.

## Introduction

Continuous EEG monitoring (CEEG) is an essential diagnostic tool to assess subclinical seizure activity or non-convulsive status epilepticus in patients with persistent or fluctuating altered mental status that are otherwise unexplained. The clinical picture of these patients can range from an awake but confused patient to a comatose patient in the intensive care unit (ICU) without any prior history of epilepsy. The EEG patterns of critically ill patients are in many ways different from those of non-critically ill patients, especially in the setting of acute brain injury. The background of EEG is slower in critically ill patients, rhythmic or periodic patterns are common, and the seizure patterns of critically ill patients also often involve a non-evolving pattern. The first definition of seizures in critically ill patients by Young et al. (1) included generalized or focal repetitive epileptiform discharges at >3 Hz lasting for >10 s. This served as a framework for current consensus definitions of electrographic seizures from the Salzburg Consensus Criteria (2, 3). This has been adopted by the American Clinical Neurophysiology Society (ACNS), and according to the updated 2021 ACNS Standardized Critical Care EEG Terminology, electrographic seizures are defined as epileptiform discharges averaging >2.5 Hz for ≥10 s or any pattern with definite evolution and lasting ≥10 s (4). If it lasts <10 s but has a clear clinical correlate, it is called an electroclinical seizure (4). The cut-off number of “10 seconds”, originally derived from observation of typical seizure duration in epilepsy patients (except for absence or myoclonic seizures) (5, 6), is rather an arbitrary number. Ictal-appearing rhythmic discharges that last under 10 s without clinical correlation are not called seizures. These discharges were first described in neonates, then later described in critically ill adults under the name, brief potentially ictal rhythmic discharges (BIRDs) (7). Since then, further efforts have been made to define, characterize, and investigate its clinical significance, which will be reviewed here. The definition of BIRDs has been modified and adopted by the 2021 ACNS Critical Care EEG Terminology. It is defined as focal or generalized rhythmic activity >4 Hz (at least six waves at a regular rate) lasting ≥0.5 to <10 s, not consistent with a known normal pattern or benign variant, not part of burst-suppression or burst-attenuation, without definite clinical correlate (see Box 1 for full definition and categories) (4).

Box 1 **Brief Potentially Ictal Rhythmic Discharges (BIRDs).**

Definition: focal (including L, BI, UI, or Mf) or generalized rhythmic activity > 4 Hz (at least six waves at a regular rate) lasting ≥0.5 to <10 s, not consistent with a known normal pattern or benign variant, not part of burst-suppression or burst-attenuation, without definite clinical correlate, and that has at least one of the following features:

a. Evolution (“evolving BIRDs,” a form of definite BIRDs)
b. Similar morphology and location as interictal epileptiform discharges or seizures in the same patient (definite BIRDs)
c. Sharply contoured but without (a) or (b) (possible BIRDs)

Note: Paroxysmal fast activity lasting ≥0.5 to <10 s qualifies as BIRDs, whether generalized (also known as generalized paroxysmal fast activity, or GPFA) or focal.

Note: Although they are termed “brief,” technically all BIRDs are “very brief” because they are <10 s.

L, lateralized, BI, Bilateral Independent, UI, Unilateral Independent, Mf, Multifocal. (Adopted from American Clinical Neurophysiology Society's Standardized Critical Care EEG Terminology: 2021 Version).

### BIRDs in neonates

Brief rhythmic discharges were first described in neonates. Because rhythmic trains of stereotyped waveforms lasting for a few seconds are a very common finding in non-specifically abnormal neonatal EEGs, a 10-s cut-off had been adopted in some neonatal studies to avoid misclassifying these waveforms as “ictal.” (8–13). However, the 10-s cut-off could also make the EEG readers disregard true ictal discharges. Shewmon discussed this problem and preferred to call them “brief ictal rhythmic discharges (BIRDs)” with an intentional ambiguity of the acronym “I” that it can either convey ictal or interictal, reflecting their conceptual fuzziness (14). He suggested that non-ictal rhythmic waveforms are generally distinguishable from true BIRDs based on (1) their common occurrence in infants with nonspecific encephalopathies and no seizures, (2) their limited range of durations in a given tracing (never longer than a few seconds), and (3) the company they keep (i.e., BIRDs tend to flock with unequivocal seizures of similar morphology) (14). Oliviera et al. tested the diagnostic and prognostic validity of BIRDs alone (i.e., when it is not accompanied by electrographic seizures) in neonates. Their study showed BIRDs by themselves were associated with a clinical history of hypoxic-ischemic encephalopathy and increased risk for the abnormal neurodevelopmental outcome and suggested including BIRDs in future studies of neonatal seizures (15). In a subsequent study by Nagarajan et al., 52 neonates were divided into three groups: (1) BERDs (here “E” stands for “EEG”) only, (2) BERDs and seizures, (3) seizures only, and found no significant difference in mortality and neurodevelopmental outcomes or background EEG impairment among these three groups and suggested that BERDs should be considered as mini seizures (16).

### BIRDs in critically ill adults

**Figure 1:**
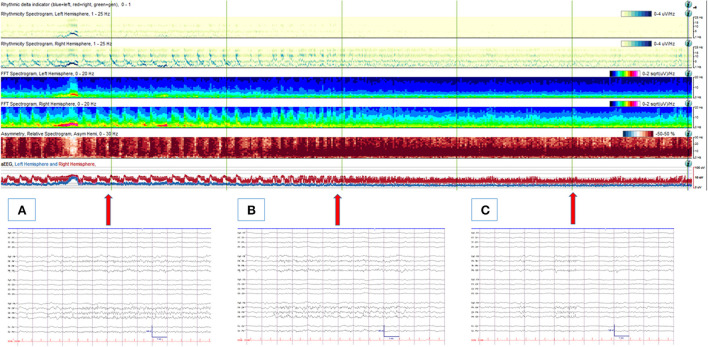
An 88-year-old man with an acute traumatic brain injury with right sided intraparenchymal hemorrhage, subdural hemorrhage and subarachnoid hemorrhage, who was noted to have frequent left facial twitching. **(Upper panel)** A quantitative EEG panel showing a total of 1-h duration. From the beginning of the record, frequent cyclic seizures are seen originating from the right hemisphere (with increased rhythmicity, power, asymmetry and amplitude from the right hemisphere with each seizure). With anti-seizure medication treatments, seizures resolve, and no more cyclic seizure patterns are depicted from the quantitative EEG analysis. **(Lower panel)** corresponding (arrows) raw EEG examples are shown. **(A)** An electrographic seizure from the right hemisphere (maximal from the parasagittal region). **(B)** Evolving BIRDs from the same area, lasting 9 s. **(C)** Non-evolving BIRDs from the same area. High- and low-pass filters were set at 1 and 70 Hz, respectively. The notch filter was off.

The occurrence of BIRDs in adults was first described in 2014 in critically ill patients. In this study, BIRDs were defined as very brief (<10 s) lateralized runs of rhythmic activity >4 Hz, with or without evolution. This study included 20 patients with BIRDs and 40 controls matched by age and the primary diagnosis. The prevalence of BIRDs was 2%. The most common frequency of BIRDs was theta (70%), typically lasting 1–3 s. In this study, none of the BIRDs showed obvious evolution. All patients with BIRDs had evidence of cerebral injury (primarily acute). The occurrence of a history of epilepsy was not significantly different from the control group. Patients with BIRDs were more likely to have seizures during CEEG than patients without BIRDs [15 of 20 (75%) vs. 10 of 40 (25%); *p* < 0.001]. Seizures often started with a morphology similar to that of BIRDs and within the same region. In all patients whose seizures were controlled with medications, BIRDs ceased after the seizures had been controlled (Figure 1). On the other hand, lateralized periodic discharges (LPDs) persisted after the seizures were controlled in most patients. Patients with BIRDs tended to have a worse outcome than controls [16 (80%) vs. 25 (63%)], but this was not statistically significant. Given the high association between BIRDs and seizures and timing of occurrence, it was strongly suggested that if BIRDs were present in the short EEG recording, continue monitoring or treat prophylactically (7). Limited by the small number of patients included in this study, further study was needed to help make the definitions more specific and help identify clinically relevant subtypes.

### BIRDs in non-critically ill patients

**Figure 2:**
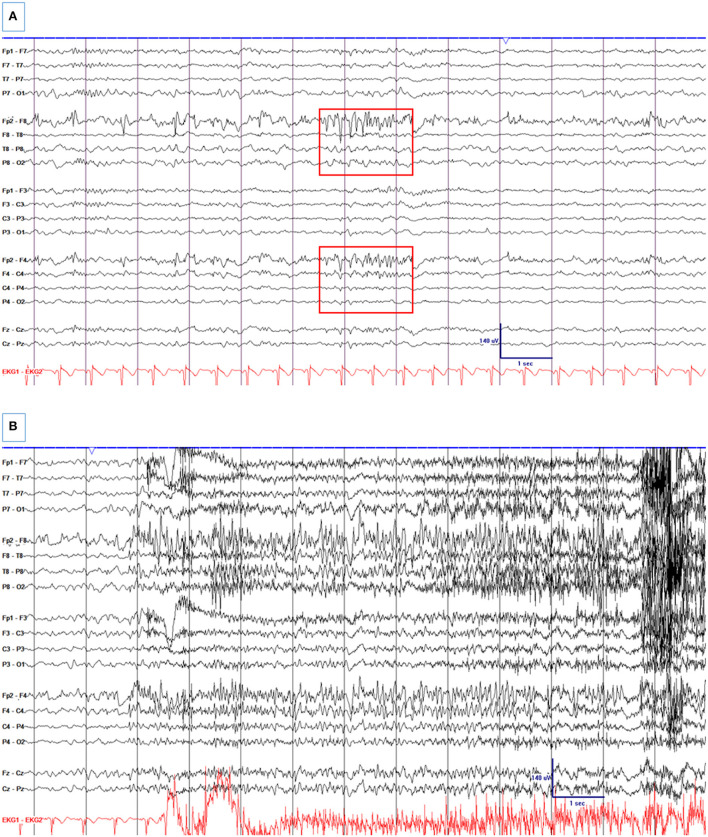
A 33-year-old woman with a remote history of traumatic brain injury at age two with a right frontal encephalomalacia and surrounding gliosis and drug-resistant epilepsy, who was electively admitted for pre-surgical evaluation. **(A)** Right frontal spikes and R frontal non-evolving BIRDs (Box 1). There is a right frontal breach rhythm. **(B)** A seizure from the right frontal region. High- and low-pass filters were set at 1 and 70 Hz, respectively. The notch filter was off.

BIRDs in non-critically ill patients were subsequently described in patients who were electively admitted to the epilepsy monitoring unit or had ambulatory EEG monitoring at home (17). In these alert and oriented patients, brief rhythmic discharges appeared with varying frequency and often in alpha or beta frequencies than theta. Generalized BIRDs were also observed and hence included in this study. BIRDs were defined as very brief (<10 s) runs of focal or generalized sharply contoured rhythmic activity >4 Hz with or without evolution. This study included 15 patients with BIRDs (1.2% prevalence) and 30 controls matched for age and etiology. Since all patients with BIRDs had epilepsy, all controls also had a history of epilepsy but no BIRDs on EEG. Patients with BIRDs were more likely to have drug-resistant epilepsy [10 of 15 (67%) vs. 5 of 30 (17%); *p* < 0.01]. The mean duration of monitoring was similar between the two groups, and seizures were captured more commonly in the BIRDs group although this did not reach the statistical significance. In this study, emphasis was made to distinguish BIRDs from normal or benign variants since many benign variants could look like BIRDs by their morphology and duration in these non-critically ill patients (e.g., mu rhythm, wicket spikes, 14- and 6-Hz positive bursts, and rhythmic mid-temporal theta discharges). One of the distinguishing features of the BIRDs from benign variants was that BIRDs were activated by sleep (especially in stage 2 sleep), whereas most benign variants are known to be present in an awake and drowsy state. However, this distinguishing feature based on the state of alertness is often not applicable to critically ill patients, especially when there are no state changes or reactivity. The location and morphology of BIRDs were also similar to the location of interictal epileptiform discharges (IEDs) or seizures in the same patient (Figure 2). Based on these features, BIRDs were defined as focal or generalized rhythmic activity >4 Hz (at least six waves at a regular rate) lasting 0.5–10 s, not consistent with a known normal pattern or benign variant, and that has at least one of the following features: (a). evolution (definite BIRDs), (b). similar morphology and location as interictal epileptiform discharges or seizures in the same patient (definite BIRDs). (c). sharply contoured but without a or b (possible BIRDs) (17). The American Clinical Neurophysiology Society's Standardized Critical Care EEG Terminology: 2021 version modified and adopted this definition and category (Box 1) (4).

### BIRDs as EEG biomarker for seizure activity and seizure onset zone

Many examples of BIRDs in the adult population could also be called paroxysmal fast activity (PFA) since it encompasses any frequencies above 4 Hz. However, with the term PFA, many of these discharges with slower frequency (i.e., theta or slower alpha frequencies) would be missed since PFA by its name means “fast.” The definition of PFA and GPFA has varied in the literature, but according to the most recent EEG term glossary, PFA is defined as “fast frequencies in the beta range or above, occurring in trains” and GPFA as “bilateral synchronous bursts of spikes of 2–10 s duration, with a frequency between 10 and 25 Hz and maximal in the frontal regions that only occurs during sleep” (18). GPFA was historically considered a marker for Lennox-Gastaut syndrome or other epileptic encephalopathies (19–21). More recently, this was recognized in patients with normal cognition and generalized epilepsy (22–25). Most recent studies have described the association between the presence of GPFA and drug resistance and no specific association with a particular type of generalized epilepsy (25, 26). Focal PFA has also been described in patients with focal epilepsy, and their relationship to seizure onset zone and intractability has been suggested (27–29). The occurrence of BIRDs and asymmetric extreme delta brush in similar regions has been reported in patients with anti-N-methyl D-aspartate (NMDA)-receptor encephalitis (30, 31). To test if different frequencies of these rhythmic discharges have any different clinical significance, different frequencies of BIRDs (including PFA), other EEG and clinical features were compared in both critically ill and non-critically ill adults (32). In this study of 94 patients with BIRDs or PFA, 74 % had epilepsy, and over half (62%) had drug-resistant epilepsy. All patients with generalized BIRDs/PFA had a history of epilepsy (67% were drug-resistant), and only 14% had epileptic encephalopathy. Sixty-six percent had seizures captured during the same recording (89% among the critically ill and 52% in non-critically ill), and the scalp EEG seizure onset zone co-localized with BIRDs/PFA in all cases, including cases with contralateral epileptiform discharges. The rate of the seizures was similar regardless of the frequency or location of the BIRDs/PFA. All patients with evolving BIRDs/PFA had electrographic seizures in the same recording, and 50% of patients with non-evolving BIRDs/PFA had seizures. In 33 patients who were in status epilepticus, when seizures resolved with anti-seizure medication treatment, BIRDs/PFA also decreased or resolved. Based on these results, BIRDs/PFA were suggested to be a biomarker for seizure activity and seizure onset zone, and since BIRDs include the frequency spectrum of PFA, it was suggested to include PFA as a sub-type of BIRDs. A recent systematic review of scalp-detected high-frequency oscillations (HFOs) in epilepsy patients reported that scalp HFOs localized the epileptogenic zone better than spikes, correlated negatively with cognition and positively with disease activity and severity, and decreased after medical and surgical treatment (33). Since no upper limit of frequency was defined in BIRDs, further study of BIRDs including scalp-detected HFOs would be valuable.

### Intracranial correlates of BIRDs

No studies thus far directly compared BIRDs to intracranial seizures. Only limited studies have attempted to systematically correlate the seizure-onset patterns on scalp EEGs with intracranial EEGs (iEEG) in epilepsy patients (34–37). A recent study compared electrocorticography (ECoG) from the responsive neurostimulation (RNS) device and simultaneous scalp EEG monitoring in drug-resistant epilepsy patients implanted with a responsive neurostimulator. In this study, the most common scalp EEG correlates for ictal-appearing long episodes that did not have scalp seizure correlates were BIRDs, including both evolving and non-evolving types (38).

Further studies, especially with simultaneous scalp EEG recordings, are needed to study the relationship between intracranial seizures and scalp EEG markers including BIRDs (both evolving and non-evolving types), to better understand the anatomical, pathological, electrophysiological, and clinical significance. It would also be interesting to study them in critically ill patients monitored with additional depth electrodes.

## Clinical use of BIRDs

To guide clinicians in assessing seizure risk, a seizure-risk scoring system (2HELPS2B) has been developed, which consists of 5 continuous EEG (CEEG) features and just one clinical variable (a history of seizure) (39). This multi-center study included 5,427 CEEG cases (>6 h) from the Critical Care EEG Research Consortium database and used a machine learning method to produce accurate, risk-calibrated scoring systems. BIRDs were seen in 3.2% of patients with a high odds ratio (18.8) and a high proportion (69%) of seizures and thus given 2 points when present. All the other variables (history of seizure, lateralized periodic discharges or rhythmic delta activity, frequency of >2 Hz for any periodic or rhythmic pattern, “plus” features, sporadic epileptiform discharges) were given 1 point. The probable seizure risk was 5% for a score of 0, 12% for 1, 27% for 2, 50% for 3, 73% for 4, 88% for 5, and >95% for a score of 6 or 7. This study was performed before the proposed categories of BIRDs (possible vs. definite, evolving vs. non-evolving) (17), so no distinction was made for these sub-categories of BIRDs. This scoring system was also shown to identify low-risk patients accurately and quickly with only 1-h screening EEG (40).

Since PFA has similar clinical significance to BIRDs (regardless of their frequencies), whether seen in critically ill patients or non-critically ill patients with epilepsy, their presence should alert the clinicians of the high likelihood of ongoing seizures or increased seizure burden of those patients. Its high correlation with seizure onset zone will also help guide the surgical planning of drug-resistant epilepsy patients, especially with further investigation of its characteristics with anatomy and pathology.

## Discussion

BIRDs are associated with a high risk of seizures and better predict the seizure onset zone compared to other interictal epileptiform discharges, thus potentially serving as a biomarker of seizure activity and seizure onset zone. The definition of BIRDs includes Paroxysmal Fast Activity (PFA), and they have similar clinical significance regardless of the exact cut-off frequencies. In patients with status epilepticus, BIRDs also resolve or decrease when seizures resolve. Thus, if BIRDs are observed on scalp EEG, longer EEG monitoring is recommended to estimate their seizure burden and to guide treatment.

EEG waveforms often appear rhythmic, and some factors make them appear sharply contoured (e.g., breach rhythm); therefore, it is essential to avoid overcalling BIRDs. In the non-critically ill, the morphology of BIRDs often resembles benign variants. So, it is important to distinguish them and not to call them BIRDs when they are, in fact, benign variants. When there are no state changes or reactivity in the critically ill, distinguishing them is challenging based on their presence in different states of alertness (41). Due to this problem, if such waveforms are seen, it is recommended to call them “possible BIRDs” in the absence of co-existing IEDs or seizures in the same patient and avoid overtreating these patterns. If the waveforms evolve, or if there are co-localizing IEDs or seizures in the same patient, then they meet the criteria for definite BIRDs, in which case they deserve treatment with anti-seizure medications. Non-evolving BIRDs also have a high correlation with seizures, so in their presence, longer monitoring is strongly suggested, and a prophylactic dose of ASMs should be considered to prevent impending seizures.

Whether to maintain or eliminate the “10-second” (clearly an arbitrary) cut-off for electrographic seizures was discussed among the authors of the critical care EEG terminology 2021 version and the Critical Care EEG Monitoring Research Consortium (CCEMRC) members both online and in person. No consensus was reached at the time as there was no convincing new literature to change it (4). Now that BIRDs are added to the official EEG terminology with the revised 2021 version of critical care EEG terminology (Box 1), with further investigations, we may be able to reach a consensus about the definition of electrographic seizures and better understand its pathologic, anatomic, and neurophysiological significance.

## Author contributions

The author confirms being the sole contributor of this work and has approved it for publication.

## Conflict of interest

The author declares that the research was conducted in the absence of any commercial or financial relationships that could be construed as a potential conflict of interest.

## Publisher's note

All claims expressed in this article are solely those of the authors and do not necessarily represent those of their affiliated organizations, or those of the publisher, the editors and the reviewers. Any product that may be evaluated in this article, or claim that may be made by its manufacturer, is not guaranteed or endorsed by the publisher.
